# Prevalence of serologic reactivity against four strains of mouse mammary tumour virus among US women with breast cancer

**DOI:** 10.1038/sj.bjc.6602977

**Published:** 2006-01-31

**Authors:** J J Goedert, C S Rabkin, S R Ross

**Affiliations:** 1 Viral Epidemiology Branch, Division of Cancer Epidemiology and Genetics, National Cancer Institute, Rockville, MD 20892, USA; 2 Department of Microbiology/Cancer Center, University of Pennsylvania School of Medicine, Philadelphia, PA 19104, USA

**Keywords:** breast cancer, mouse mammary tumour virus (MMTV), seroprevalence, Western immunoblot, immunoprecipitation

## Abstract

Mouse mammary tumour virus (MMTV) causes breast cancer in mice, and MMTV-specific antibodies develop to high titers among mice infected as adults. Whether MMTV or a related virus infects humans is uncertain, because MMTV DNA sequences have been detected inconsistently and because serologic methods have varied widely. The current study used immunoblot and immunoprecipitation with four strains of MMTV (RIII, FM, C3H, and LA) to detect specific antibodies in 92 sera from US women with breast cancer and in masked dilutions of monoclonal hybridoma and hyperimmunised goat positive-control reagents. In these positive controls, MMTV antibodies of the expected molecular weights were detected at high titer (1 : 100 in the monoclonal reagent, 1 : 10000 in the hyperimmunised goat serum). Nearly 30% of the sera from women with breast cancer had at least one faint band on an immunoblot, but none of these matched the molecular weight of bands revealed by probing the same blot strips with the goat serum. The goat serum readily immunoprecipitated MMTV antigens from all four strains of MMTV, but MMTV antigens were not immunoprecipitated by any of the six breast cancer sera that had four or more nonspecific immunoblot bands. Thus, among women with breast cancer, we found no MMTV-specific antibodies. The upper 95% confidence limit implies that MMTV seroprevalence among breast cancer patients does not exceed 3%.

The mouse mammary tumour virus (MMTV), a betaretrovirus, causes breast cancer in mice. During the past 10 years, molecular evidence of MMTV in human breast cancer tissue has been reported by a few laboratories using polymerase chain reaction (PCR) techniques (summarised in Holland and Pogo (2004)). These findings are controversial, because other laboratories have been unable to replicate detection of MMTV and because the DNA amplified by PCR in some laboratories has had homology with nonviral sequences in the human genome (summarised in Mant *et al* (2004)). Serologic studies to identify MMTV antibodies complement these PCR-based molecular studies of breast cancer tissue. During the late 1970s and early 1980s, detection of serum antibodies against MMTV-infected cells or proteins from these cells among women with breast cancer provided support for the possibility of a human homologue of MMTV. There was, however, substantial heterogeneity in methods, in antigens recognised by the sera, and in seroprevalence associations. The results and limitations, particularly with respect to specificity, of these early studies were reviewed by Dion *et al* (1987). By Western immunoblot with disrupted, purified milk-borne MMTV of the RIII strain, Dion and co-workers found no antibodies against MMTV viral antigens in 1 : 5 dilutions of sera from 30 breast cancer patients or 30 control patients (Dion *et al*, 1987). In 1 : 8 dilutions of sera in enzyme immunoassays, Dion *et al* found modestly higher reactivity against column-purified p18 from MMTV but not against four other MMTV column-purified proteins or glycoproteins in breast cancer patients compared to controls (Dion *et al*, 1987). Among 300 Czech subjects (90 healthy controls, 60 with breast cancer, and 150 with other malignancies or serious diseases) whose sera, diluted 1 : 100, were tested by immunoblot heavily loaded with proteins and glycoproteins from the GR/N strain of MMTV, Kovarik *et al* found frequent reactivity against a 42 kDa cellular contaminant of the virus, but few with reactivity against viral antigens and no differences between cases and controls (Kovarik *et al*, 1989). All of the previously published studies used only a single strain of MMTV. We, therefore, sought to estimate the MMTV seroprevalence among US women with breast cancer by using a wider variety of MMTV strains. We used both immunoblotting and immunoprecipitation to maximise specificity of the anti-MMTV reactivity.

## MATERIALS AND METHODS

### Patients and controls

Between 1979 and 1988, the Immunodiagnosis Serum Bank collected, separated, and stored frozen sera from patients at the Mayo Clinic who had common malignancies (Dimagno *et al*, 1989). To estimate MMTV seroprevalence, sera from 92 women with breast cancer in this bank were selected, based on total number of available aliquots and availability of at least one 0.25 ml aliquot. Data on age at blood draw, histologic diagnosis, tumour stage, cancer treatment history, and smoking status were available. Identifying data had been removed. Exemption from Institutional Review Board review was obtained from the NIH Office of Human Subjects Research. Monoclonal *α*-gp52 hybridoma supernatant BL6 5D, as well as caprine sera from goats hyperimmunised with MMTV (*α*-MMTV) or with gp52 (polyclonal *α*-gp52), served as positive control reagents (Dzuris *et al*, 1999; Purdy *et al*, 2003). In a central repository, three aliquots of each of these reagents (neat, 1 : 10 and 1 : 100 dilutions in defibrinated plasma (Basematrix, BBI-Biotech Inc., Frederick, MD, USA)) were put into vials identical to the human sera. The nine positive controls were interspersed among the 92 human sera and could not be identified by the laboratory. To assess the integrity of the sera, they were tested for rubella antibodies with a commercial agglutination test (BD Biosciences Inc.).

### Detection of MMTV-specific antibodies

Immunoblot analysis was performed as described previously (Selmi *et al*, 2004). Briefly, total protein was extracted from virions purified by pelleting through sucrose cushions, as previously described (Le Bon *et al*, 1999). The virions were purified from mammary tumours of C3H/HeN mice infected with MMTV (FM) or MMTV (LA), milk from RIII mice (MMTV (RIII); a gift from Akhil Vaidya), or supernatants from the cultured mammary tumour cell line Mm5MT (MMTV (C3H)). Virions were disrupted in lysis buffer containing 25 mM Tris-HCl (pH 8.0), 150 mM NaCl, 2 mM EDTA, 0.1% sodium dodecyl sulfate, 0.5% Nonidet P-40, and 0.5% deoxycholate. The proteins were separated by sodium dodecyl sulfate–10% polyacrylamide gel electrophoresis (SDS–PAGE) and transferred to nitrocellulose. As the virus preparations varied in purity, we normalised the amounts of virus electrophoresed in each lane by initially running different dilutions of the virus preparations and probing the filters with goat anti-MMTV serum, as described below.

Sera were kept frozen at or below −70°C until testing. Each masked serum specimen was diluted 1 : 100 in phosphate-buffered saline (PBS) containing 0.1% Tween-20 (PBS-Tween) and 5% nonfat dried milk, incubated for 5 h with the blots and then washed extensively with PBS-Tween. As the primary sera were masked, we used a mixture of horseradish peroxidase (HRP)-conjugated donkey anti-human (1 : 8000; Jackson Immunoresearch Inc., West Grove, PA), rabbit anti-goat (1 : 5000; Sigma Inc., St. Louis, MO) and sheep anti-mouse (1 : 5000; Amersham Inc., Piscataway, NJ) secondary antibodies to detect bound primary antibodies. This mixture was added to each blot in PBS-Tween-milk, incubated for 30 min and again washed extensively with PBS-Tween. To ensure that the mixture of secondary antibodies was species-specific, we also blindly tested 22 of the samples with the anti-human secondary antibody alone and found that the same pattern of bands seen with the mixture, except for two samples that were masked, diluted goat serum. These latter two samples showed no bands on the virus blots probed with the human secondary antibody alone but showed the typical MMTV pattern when the mixture of secondary antibodies was used (not shown). The blots were developed with ECL Enhanced Western Blotting Detection Reagent, according to the manufacturer's instructions (Amersham Inc.) To characterise whether antibodies revealed in the immunoblots had similar molecular weight to those in caprine sera, blot strips that demonstrated antibody bands were reprobed with polyclonal goat anti-MMTV antisera (*α*-MMTV, 1 : 3000) followed by the HRP-conjugated rabbit anti-goat secondary antibody. Photographs of the blots with human and *α*-MMTV sera were aligned and overlaid for visual comparison. Side-by-side comparisons are provided. To maximise detection, the human blots were exposed for up to 5 min.

For the immunoprecipitation experiment, approximately 50 *μ*g of purified protein from each of the four MMTV strains in 200 *μ*l final volume of RIPA buffer (25 mM Tris, pH 7.8, 150 mM NaCl, 2 mM EDTA, 0.5% NP40, 0.5% deoxycholate, 0.1% sodium dodecyl sulphate, 1 mM PMSF, 5 *μ*g ml^−1^ leupeptin, 5 *μ*g ml^−1^ aprotinin, 2 *μ*g ml^−1^ pepstatin A) were added to 1 *μ*l of human serum, or to 1 *μ*l of a 1 : 10 dilution of the polyclonal goat anti-MMTV serum followed by 50 *μ*l of Protein G sepharose (Invitrogen Inc., Carlsbad, CA) and incubated overnight at 4°C. After extensive washing, the proteins were eluted in 50 *μ*l of SDS–PAGE sample buffer and 10 *μ*l were electrophoresed on SDS–polyacrylamide gels. The proteins were then transferred to nitrocellulose. For the human sera, Western blot analysis using polyclonal goat anti-MMTV and rabbit anti-goat antibodies was carried out as described in the preceding paragraph. For the control immunoprecipitation performed with the goat anti-MMTV antisera, the Goat TrueBlot Western Blot Kit (eBioscience Inc., San Diego, CA) was used for detection of the viral proteins to prevent obscuring of MMTV bands by the goat heavy and light chain immunoglobulin used in the immunoprecipitation.

## RESULTS

In 65 (71%) of the 92 sera from women with breast cancer, no band was seen in any of the four MMTV immunoblots. One serum demonstrated a smear pattern in three of the four MMTV immunoblots and could not be evaluated. Of the other 26 sera, across all four MMTV immunoblots, 14 (15%) had one band; two (2%) had two bands; four (4%) had three bands; three (3%) had four bands; two (#36 and 60) had six bands; and one (#16) had eight bands (Table 1). Age, breast cancer histology, breast cancer stage, history of cancer treatment, and smoking status did not differ between women whose sera had multiple bands or one band and those who sera had no band (data not presented).

**Figure 1:**
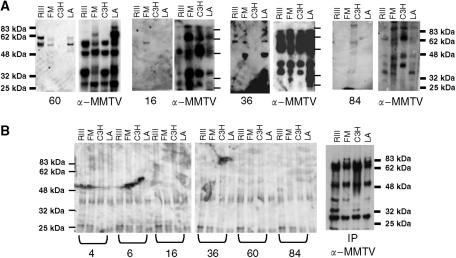
MMTV antibody immunoblots and immunoprecipitation with human and goat sera. (**A**) (upper panel). Each human serum was tested by western immunoblot for antibodies against antigens in four strains of MMTV (RIII, FM, C3H, and LA). Four sera (#60, 16, 36, and 84) demonstrating bands are presented. Each blot was then stripped and re-probed with hyperimmunised goat antiserum (labeled *α*-MMTV). Although various bands are present with each of these selected human sera (e.g., two bands against the RIII, FM, and LA strains in #36 and 60, four bands against the C3 H strain in #84), none matched the molecular weight of the bands revealed with the *α*-MMTV positive control serum. (**B**) (lower panel). Immunoprecipitation of antigens in four strains of MMTV (RIII, FM, C3H, and LA) with six human sera and with hyperimmunised goat antiserum (*α*-MMTV). MMTV antigens precipitated only with the *α*-MMTV positive control serum.

There was heterogeneity in the frequencies of serologic reactivity across the four MMTV strains. Considering all reactivity of all sera (except the one with a smear pattern), there were 19 bands against MMTV strain RIII; 20 bands against strain FM; 18 bands against strain LA; and only five bands against strain C3H. Serum #60 had two distinct bands of approximate molecular weight 56 and 60 kDa against each strain except C3H, and these differed from the bands that were detected with the *α*-MMTV positive control serum (Figure 1A). Serum #16 had one or more bands against all four strains, all of which were very faint compared with the *α*-MMTV positive control serum (Figure 1A). None of the bands, including those in sera #60 and #16, had the same molecular weight as the *α*-MMTV positive control serum on the same blot strips (Figure 1A).

In contrast, the masked dilutions of the three positive control reagents performed as expected. The caprine *α*-MMTV and polyclonal *α*-gp52 reagents were detected at the final dilution, 1 : 10000; further dilutions were not tested. The monoclonal hybridoma supernatant containing *α*-gp52 antibodies was detected at 1 : 100 but not at 1 : 1000 or 1 : 10000. No antibody bands were seen in an unmasked human negative control serum. Rubella antibodies were detected in 85 (92%) of the 92 human sera. Rubella antibodies were not detected in the three neat caprine and monoclonal reagents, but they were detected in the reagents that had been diluted with human plasma-derived Basematrix.

Immunoprecipitation assays were performed with the sera that had four or more bands on immunoblots to determine whether the reactivity was directed against MMTV proteins. As shown in Figure 1B, these six sera (#4, 6, 16, 36, 60, and 84) had faint background staining with prolonged exposure (20 min), most likely due to proteins present in the human sera, since they occurred with virus partially purified from different sources (milk (RIII), tumour tissue (FM and LA), and cultured cells (C3H)). Importantly, the human sera did not immunoprecipitate MMTV-specific antigens from any of the four strains. In contrast, MMTV antigens were readily precipitated with the diluted goat *α*-MMTV positive control serum (5 s exposure). These data indicate that the bands detected by the human serum samples were not MMTV viral proteins.

## DISCUSSION

We found that nearly 30% of sera from US women with breast cancer had some serologic reactivity against protein in one or more of four different MMTV strains. However, none of this reactivity was specifically directed against MMTV antigens. This reactivity could be due to contaminating cellular or milk proteins present in the partially purified virion preparations. Alternatively, the MMTV envelope, like all retroviruses, incorporates many nonviral, cellular proteins as it is released from the cell. These nonviral proteins can be immunogenic, as illustrated by the protection of macaques against simian immunodeficiency virus (SIV) by vaccination with cells that did not contain SIV (Stott, 1991). Given the similarities across mammalian species, it is not surprising that some women would have developed antibodies against one or more nonviral proteins in the preparations of MMTV that we used. While it is possible that some of the women with reactive sera were exposed to mice, it is more likely that the antibodies we detected were cross-reactive with other cellular antigens, possibly including auto-antibodies (Tax and Manson, 1987; Kovarik *et al*, 1989). Development of antibodies against nonspecific autoantigens occurs frequently among women with breast cancer (Coronella-Wood and Hersh, 2003).

Mice infected as adults that develop MMTV-induced mammary cancers have life-long, high-titer antibodies against both MMTV and heterophile antigens, (Bowen *et al*, 1976) whereas mice infected as neonates generally have only a transient antibody response (Luther *et al*, 1997; Purdy *et al*, 2003). Seronegative MMTV infection of newborn humans resulting in breast cancer is unlikely, because breast cancer risk is not increased among women who were themselves breast-fed (Titus-Ernstoff *et al*, 1998). Thus, if MMTV infects humans, antibodies should be detected. Unlike sera from mice infected as adults, none of 91 sera from our women with breast cancer had specific, much less high titer, antibodies against MMTV. The upper 95% confidence limit for 0/91 implies that MMTV seroprevalence among women with breast cancer does not exceed 3%.

Antibodies should be present if genes that code for MMTV envelope or other structural proteins are expressed in humans. There are, however, no published reports of anti-MMTV seropositivity among breast cancer patients in whom MMTV RNA expression was found, nor among those in whom DNA sequences resembling MMTV were found (Holland and Pogo, 2004). Likewise, there are no reports of MMTV DNA detection among such patients who were putatively MMTV seropositive. One laboratory, using PCR-based approaches, has reported detection of MMTV-like DNA in liver biopsies and lymph nodes, as well as MMTV-like RNA in serum (Mason *et al*, 2004). Moreover, they found anti-MMTV antibodies in seven of nine patients with primary biliary cirrhosis (Mason *et al*, 2004). In that study, MMTV antibodies were detected by immunoblot analysis of extracts made from the Mm5MT cell line, the same cells from which we purified the MMTV (C3H) virions, and they used the same polyclonal goat *α*-MMTV antisera for a positive control. However, in a much larger series of primary biliary cirrhosis patients, a different laboratory was unable to detect MMTV-like DNA in either liver biopsies or peripheral blood mononuclear cells by PCR or antibodies against three different strains of MMTV, including the C3H strain (Selmi *et al*, 2004). The human endogenous retrovirus (HERV) K10 has substantial homology with MMTV, and specific antibodies can be detected when HERV K10 envelope or core (gag) is expressed, as occurs in some cases of testis cancer (Goedert *et al*, 1999). Mant *et al* (2004) have suggested that the MMTV-like DNA sequences detected in humans are not MMTV or HERV K10 but rather are another homologous region of the human genome.

Our study has several weaknesses and strengths. The sera that we evaluated were collected 15–20 years earlier, during which time they might have deteriorated. However, storage of this collection at or below −70°C for up to 10 years was shown to have negligible effect on serum chemistry values other than bilirubin and creatinine (Dimagno *et al*, 1989). Moreover, some 15 years after they were collected, sera from this collection were proven to contain antibodies against human papilloma virus type 16 and adeno-associated virus (Strickler *et al*, 1998; Strickler *et al*, 1999). More directly, in the current study, we found that 92% of the sera that we tested had rubella antibodies, as expected for women born before 1960. The sera were collected from one large center in the US, were accompanied by limited clinical data, and are not necessarily representative of all US women with breast cancer. To strengthen our study, among the 92 sera from women with breast cancer, we interspersed dilutions of masked positive control sera. These controls demonstrated that our methods could detect MMTV antibodies in masked goat sera and *α*-gp52 monoclonal hybridoma supernatant. To maximise sensitivity for detecting anti-MMTV in human sera, we used four purified MMTV immunoblot preparations, as well as prolonged exposure times. To maximise specificity of the anti-MMTV reactivity, we used immunoprecipitation, as well as overlaying immunoblots probed with caprine *α*-MMTV to directly compare the number, strength, and molecular weights of immunoblot bands found with human sera. In contrast to the *α*-MMTV positive control serum, the human sera were negative or had only weak, nonspecific reactivity.

In summary, we found no evidence of antibodies against MMTV among US women with breast cancer.

## Figures and Tables

**Table 1:** Prevalence of antibody reactivity against four strains of MMTV among women with breast cancer in the United States

**Total bands^a^**	**No. (%) of women**	**Median (range) age**
More than 4	3 (3)	47 (37–53)
4	3 (3)	48 (41–61)
3	4 (4)	49 (40–58)
2	2 (2)	71 (55–86)
1	14 (15)	53 (35–71)
0	65 (71)	57 (33–95)
Smear	1 (1)	73

a Sum of all discrete bands across the immunoblots of all four MMTV strains (RIII, FM, C3H, and LA). As shown in Figure 1A, serum from Patient #16 had eight bands (1 against RIII, 2 against FM, 2 against C3H, and 3 against LA), and sera from Patients #36 and 60 had six bands (two each of the same molecular weight against RIII, FM, and LA).
